# Higher toxicity with 42 Gy in 10 fractions as a total dose for 3D-conformal accelerated partial breast irradiation: results from a dose escalation phase II trial

**DOI:** 10.1186/1748-717X-7-141

**Published:** 2012-08-22

**Authors:** Celine Bourgier, Catalina Acevedo-Henao, Ariane Dunant, Christine Rossier, Antonin Levy, Mohamed El Nemr, Isabelle Dumas, Suzette Delaloge, Marie-Christine Mathieu, Jean-Remi Garbay, Alphonse Taghian, Hugo Marsiglia

**Affiliations:** 1 Department of Radiation Oncology, Institut Gustave Roussy, 114 rue Edouard Vaillant, 94 805, Villejuif, France; 2 Biostatistics and Epidemiology Unit, Institut Gustave Roussy, Villejuif, France; 3 Alexandria University, Alexandria, Egypt; 4 Physics Unit, Department of Radiation Oncology, Institut Gustave Roussy, Villejuif, France; 5 Department of Breast Oncology, Institut Gustave Roussy, Villejuif, France; 6 Department of Pathology, Institut Gustave Roussy, Villejuif, France; 7 Department of Breast Surgery, Institut Gustave Roussy, Villejuif, France; 8 Department of Radiation Oncology, Massachusetts General Hospital, Harvard Medical School, Boston, MA, USA; 9 Grupoimo, Madrid, Spain

**Keywords:** 3D-conformal accelerated partial breast irradiation, Dose escalation

## Abstract

**Objective:**

Recent recommendations regarding indications of accelerated partial breast irradiation (APBI) have been put forward for selected breast cancer (BC) patients. However, some treatment planning parameters, such as total dose, are not yet well defined. The Institut Gustave Roussy has initiated a dose escalation trial at the 40 Gy/10 fractions/5 days and at a further step of total dose (TD) of 42 Gy/10 fractions/ 5 days. Here, we report early results of the latest step compared with the 40 Gy dose level.

**Methods and materials:**

From October 2007 to March 2010, a total of 48 pT1N0 BC patients were enrolled within this clinical trial: 17 patients at a TD of 42 Gy/10f/5d and 31 at a TD of 40 Gy/10f/5d. Median follow-up was 19 months (min-max, 12–26). All the patients were treated by APBI using a technique with 2 minitangents and an “enface” electrons delivering 20% of the total dose. Toxicities were systematically assessed at 1; 2; 6 months and then every 6 months.

**Results:**

Patients’ recruitment of 42 Gy step was ended owing to persistent grade 3 toxicity 6 months after APBI completion (n = 1). Early toxicities were statistically higher after a total dose of 42 Gy regarding grade ≥2 dry (p = 0.01) and moist (p = 0.05) skin desquamation. Breast pain was also statistically higher in the 42 Gy step compared to 40 Gy step (p = 0.02). Other late toxicities (grade ≥2 fibrosis and telangectasia) were not statistically different between 42 Gy and 40 Gy.

**Conclusions:**

Early toxicities were more severe and higher rates of late toxicities were observed after 42 Gy/10 fractions/5 days when compared to 40 Gy/10 fractions/5 days. This data suggest that 40 Gy/10 fractions/ 5 days could potentially be the maximum tolerance for PBI although longer follow-up is warranted to better assess late toxicities.

## Introduction

The current trend in early breast cancer (BC) is to shorten overall treatment time either by hypofractionated whole breast irradiation [1] or by accelerated partial breast irradiation (APBI). Local control efficacy of 3D-conformal APBI compared to whole breast irradiation is currently investigated through large clinical trials, such as NSABP B-39/ RTOG-0413 phase III trial [2] and RAPID trial [3]. Waiting for definitive results of those clinical trials, the American and European Radiation Oncology Societies (ASTRO and ESTRO) put forward guidelines for selected group of patients with breast cancer at low risk of local relapse for whom APBI could be performed out of clinical trials [4,5]. Even though 3D-conformal APBI is one of the most used APBI technique due to its practicality [6], the optimal total dose and fractionation is not yet well defined. A dose escalation 3D-conformal APBI trial has been initiated in 2003 at the Massachussetts General Hospital (MGH) to assess the optimal total dose consisting in delivering 32 Gy/8fractions (f) over 4 days; 36 Gy/9f over 4½ days; 40 Gy/10f over 5 days (“40 Gy step”). In collaboration with the MGH, we extended this clinical trial by the enrolment of patients in the 40 Gy step [7] and in an additional and further step : 42 Gy in 10 BID fractions over 5 days. We have recently reported the 40 Gy step ((7)) and here, we report early results of the latest step comparing 42 Gy/10 f with the 40 Gy/10 f dose level.

## Methods and materials

### Study population

From October 2007 to March 2010, 48 pT1N0 unifocal BC patients were prospectively enrolled in an institutional and national review board-approved phase II trial. Patients eligibility has been already described in [7] and they were treated 4 to 12 weeks after a breast conservative surgery with 3D-conformal APBI.

Stopping rules were any persistent and severe (grade 3) toxicities (fibrosis or fat necrosis) or persistent early grade 3 toxicity occurring during the year that followed APBI completion. 31 breast cancer patients were enrolled in the 40 Gy step; and only 17 breast cancer patients in the 42 Gy step. Hence, per the trial stopping rules, patients’ recruitment was ended owing to persistent grade 3 toxicity 6 months after APBI completion (n = 1).

### Treatment planning and toxicity assessment

Briefly, all patients underwent to computed tomography (CT)-based 3D planning. The ipsilateral and contralateral breast, left and right lungs and heart were contoured [8]. The clinical target volume (CTV) was defined as the delineation of the visible lumpectomy cavity and the surgical clips placed inside the lumpectomy cavity [9-11]; planning target volume (PTV) as a CTV expansion of 2.3 to 2.8 cm. The PTV for evaluation (PTV_EVAL) was the PTV with exclusion of the anterior chest wall/pectoralis muscles and the first 5 mm of tissue under the skin and anterior chest wall/pectoralis muscles (according to the definition by Vicini et al. [12]). The technique used was a combination of photon beams of 6 MV and electrons of 6 to 22 MeV. The dosimetric plan was performed using Dosigray 4.1.2.50 TPS (Dosisoft) [11]. Normal tissue dose–volume constraints were defined as followed: 50% of the non-targeted breast volume had be less than 50% of the prescribed dose; the PTV_EVAL-to-whole breast ratio had to be less than 25%; and a limited dose was to be received by heart and lungs. The lung volume dose constraints used were as follows: <3% at 20 Gy, <10% at 10 Gy, and <20% at 5 Gy [13]. Two successive total doses were prospectively assessed: 40 Gy in 10 BID fractions over 5 days and 42 Gy in 10 BID fractions over 5 days, with a minimum interfraction interval of 6 hours.

### Statistical methods

Patients and tumors characteristics were compared with the Wilcoxon test between each steps (40 Gy and 42 Gy). Dosimetric data of 40 Gy and 42gy steps were compared with the Wilcoxon test.

Initial toxicities assessment was performed before APBI; then 1, 2, 6 months after APBI completion and further every 6 months. Toxicities were scored according to the RTOG scale: grade 0 for none; grade 1 for mild; grade 2 for moderate; grade 3 for severe; and grade 4 for fatal toxicities. Toxicities incidences (by type and global), estimated for each of the 2 total doses, were plotted with Kaplan Meier incidence curves and compared with a log-rank test.

## Results

Patients and tumor characteristics are listed in Table 1. Median follow-up was 19 months (min-max, 12–26) in the 42 Gy step vs 32 months (min-max, 23–40) in the 40 Gy step. Mean age was 67 years (min-max, 52–76). Median tumor size was 10 mm (min-max, 4–20). Enrolled patients had mainly pT1N0 invasive ductal carcinoma (82%), positive hormone receptors, negative Her2 overexpression.

**Table 1:** Patients and tumor characteristics (Abbreviation: ER = estrogen receptor; PR = progesteron receptor)

	**42 Gy step (n = 17)**	**40 Gy step (n = 31)**
Median age (years)(min-max)	67 (52–76)	65 (53–79)
Median tumor size (mm) (min-max)	10 (4–20)	11 (5–20)
Histology		
Invasive ductal carcinoma (IDC)	82%	90%
Mucinous/tubular/other invasive carcinoma	6%	-
Ductal Carcinoma In Situ (DCIS)	12%	10%
Nodal status		
pN0	100%	100%
Scarff & Bloom histology grade		
1	44%	64%
2	38%	32%
3	13%	4%
ER status		
Positive	100%	100%
PR status (+)		
Positive	69%	77%
Negative	31%	23%
Her2 status		
Negative	100%	100%

### Planning target volume and normal tissue dosimetry

PTV_EVAL coverage was adequate with a mean dose to the PTV_EVAL at 43.7 Gy (range, 43.1 – 44.4 Gy). Mean breast V20Gy was at 41.6% (range, 15.4% – 55.2%). Mean non-target breast V42Gy and V20Gy were at 6% (range, 1.6%–11.9%) and 30.9% (range, 12.5%-44.5%), respectively. Mean ipsilateral lung dose was 1.4 Gy (range, 1.0–1.8 Gy), and the V20 Gy was 0.2% (range, 0.0% – 1.0%). The mean heart dose was 1.1 Gy (range, 1.0–1.3 Gy). No significant difference was seen between dosimetric parameters of 40 and 42 Gy steps except for a higher V5Gy in the heart in the 40 Gy step (*p* = 0.05). Other dosimetric characteristics are listed in Table 2.

**Table 2:** Dosimetric characteristics at the 42 Gy and 40 Gy step

**Characteristic**	**42 Gy (n = 17)**		**40 Gy (n = 31 patients, 32 tumors)**	
	**Median**	**Min - max**	**Median**	**Min - max**
PTV coverage				
V100%	99	97 - 100	99	95 - 100
V95%	100	100 - 100	100	99 - 100
V90%	100	100 - 100	100	99 - 100
Ipsilateral breast				
V32 Gy (%)	35	13 - 48	40	21 - 50
V24 Gy (%)	39	15 - 53	46	23 - 54
V16 Gy (%)	43	16 - 57	49	24 - 57
V8 Gy (%)	54	18 - 68	55	26 - 75
Non target breast				
V40 Gy (%)	10	3 - 18	8	3 - 18
V32 Gy (%)	25	10 - 35	29	6 - 38
V20 Gy (%)	32	12 - 44	35	8 - 45
V10 Gy (%)	44	14 - 60	44	11 - 63
Ipsilateral lung				
V20 Gy (%)	0	0 - 1	0	0 - 1
V10 Gy (%)	0	0 - 2	1	0 - 6
V5 Gy (%)	3	0 - 5	4	0 - 12
Heart				
V20 Gy (%)	0	0 - 0	0	0 - 0
V10 Gy (%)	0	0 - 0	0	0 - 1
V5 Gy (%)	0	0 - 1	1	0 - 4
left breast cancer, n = 9			left breast cancer, n = 13	

### Early side effects for 42 Gy cohort (Table 3 and Figure 1)

**Table 3:** Early and late toxicity

			**M1**	**M2**	**M6**	**M12**	**M18**	**M24**
Early toxicities								
Erythema	42 Gy		(n = 17)	(n = 17)	(n = 17)	(n = 17)	(n = 17)	(n = 17)
		Grade 0	3	7	17	16	17	17
		Grade 1	7	3	-	1	-	-
		Grade 2	4	6	-	-	-	-
		Unknown	3	1	-	-	-	-
	40 Gy		(n =31)	(n =31)	(n = 31)	(n = 31)	(n = 31)	(n = 31)
		Grade 0	10	10	26	31	31	31
		Grade 1	13	18	5	-	-	-
		Grade 2	8	3	-	-	-	-
		Unknown	-	-	-	-	-	-
Dry desquamation	42 Gy		(n = 17)	(n = 17)	(n = 17)	(n = 17)	(n = 17)	(n = 17)
		Grade 0	10	11	16	17	17	17
		Grade 1	2	1	1	-	-	-
		Grade 2	2	4	-	-	-	-
		Unknown	3	1	-	-	-	-
	40 Gy		(n =31)	(n =31)	(n = 31)	(n = 31)	(n = 31)	(n = 31)
		Grade 0	19	26	31	31	31	31
		Grade 1	4	3	-	-	-	-
		Grade 2	2	-	-	-	-	-
		Unknown	6	2	-	-	-	-
Moist desquamation	42 Gy		(n = 17)	(n = 17)	(n = 17)	(n = 17)	(n = 17)	(n = 17)
		Grade 0	13	15	17	17	17	17
		Grade 1	-	-	-	-	-	-
		Grade 2	1	1	-	-	-	-
		Unknown	3	1	-	-	-	-
	40 Gy		(n =31)	(n =31)	(n = 31)	(n = 31)	(n = 31)	(n = 31)
		Grade 0	24	28	31	31	31	31
		Grade 1	1	1	-	-	-	-
		Grade 2	-	-	-	-	-	-
		Unknown	6	2	-	-	-	-
Late toxicities								
Fibrosis or retraction	42 Gy		(n = 17)	(n = 17)	(n = 17)	(n = 17)	(n = 17)	(n = 17)
		Grade 0	17	17	6	4	1	-
		Grade 1	-	-	7	10	6	5
		Grade 2	-	-	3	2	3	1
		Grade 3	-	-	1	1	-	-
		Unknown	-	-	-	-	7	11
	40 Gy		(n =31)	(n =31)	(n = 31)	(n = 31)	(n = 31)	(n = 31)
		Grade 0	31	30	23	19	9	8
		Grade 1	-	1	5	10	15	19
		Grade 2	-	-	2	1	3	2
		Grade 3	-	-	-	1	-	-
		Unknown	-	-	1	-	4	2
Breast pain	42 Gy		(n = 17)	(n = 17)	(n = 17)	(n = 17)	(n = 17)	(n = 17)
		Grade 0	9	6	6	5	3	2
		Grade 1	5	10	7	9	6	2
		Grade 2	-	-	3	2	3	2
		Grade 3	-	-	1	1	-	-
		Unknown	3	1	-	-	5	11
	40 Gy		(n =31)	(n =31)	(n = 31)	(n = 31)	(n = 31)	(n = 31)
		Grade 0	11	14	17	16	12	12
		Grade 1	11	13	11	10	10	12
		Grade 2	3	2	2	5	4	5
		Grade 3	-	-	-	-	-	-
		Unknown	6	2	1	-	5	2
Telangiectasia	42 Gy		(n = 17)	(n = 17)	(n = 17)	(n = 17)	(n = 17)	(n = 17)
		Grade 0	17	17	15	12	7	4
		Grade 1	-	-	-	-	2	1
		Grade 2	-	-	1	3	2	-
		Grade 3	-	-	1	2	1	1
		Unknown	-	-	-	-	5	11
	40 Gy		(n =31)	(n =31)	(n = 31)	(n = 31)	(n = 31)	(n = 31)
		Grade 0	29	30	29	25	19	17
		Grade 1	1	-	-	3	3	6
		Grade 2	1	1	1	2	5	6
		Grade 3	-	-	-	-	-	-
		Unknown	-	-	1	1	4	2

**Figure 1:**
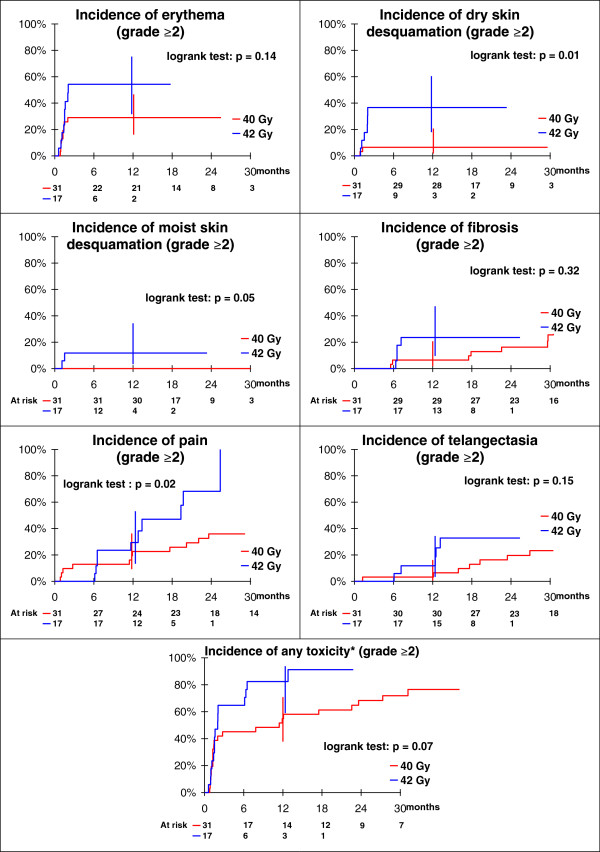
Incidence of grade ≥2 toxicities

Although patients cohort is smaller than those in 40 Gy step, patients had more grade 2 erythema two months after APBI completion (n = 6/17; p = 0.14), but was not statistically significant when compared to 40 Gy step. On the contrary, patients who were enrolled in the 42 Gy step had statistically more grade 2 moist desquamation (p = 0.05); and had statistically more grade 2 dry desquamation (p = 0.01) at early time points (1 and 2 months after APBI completion).

Late toxicities increased with higher total dose and occurred earlier (at 6 months). Grade ≥ 2 telangiectasia were observed at 6 months in 2/17 (12%) of patients in the 42 Gy versus 1/31 (3.2%) for the 40 Gy and increased with time (30% in the 42 Gy versus 6% for the 40 Gy at 18 months). Grade ≥ 2 breast fibrosis was observed in 2/31 (6.5%) of 40 Gy-patients and in 4/17 (24%) of 42 Gy-patients at 6 months. Similarly, grade ≥ 2 breast fibrosis was reported at 18 months in 3/31 (10%) of 40 Gy-patients and in 3/17 (18%) of 42 Gy-patients. Hence, cosmetic results assessed by physicians were considered as fair in 3/13 patients at 18 months in the 42 Gy step whereas in the 40 Gy step was 2/26 patients. However grade ≥2 toxicities between 40 Gy and 42 Gy steps were not statistically different except for breast pain (p = 0.02). Patients treated at the 42 Gy step indeed observed more grade ≥2 breast pain (3/17 patients) rather than those treated at the 40 Gy step (4/31 patients), the difference was statistically significant (p = 0.02).

## Discussion

A higher total dose of 42 Gy delivered by 3D-conformal APBI showed adequate treatment planning and did not increase organs at risk exposure when compared to other 3D-conformal APBI using lesser total dose [7]. Early tolerance was less suitable after a total dose of 42 Gy with higher risk of dry or moist skin desquamation. In addition, even though patients’ enrollment was ended owing to persistent grade 3 toxicity 6 months after APBI completion, all late grade ≥2 toxicities were not statistically different between 40 and 42 Gy steps except for breast pain.

Many APBI clinical trials are currently in progress using 3D-conformal radiotherapy and using different total doses and fractionations. A dose escalation phase II trial has been initiated at IGR in 2007 to assess the maximal dose tolerance.

Biologic effects in terms of tumor control and normal tissue reactions are estimated through biologically effective dose (BED) values. The BED values for APBI schedules should be interpreted by taking into account that the linear quadratic model is not enough adequate to accurately assess accelerated and bifractionated irradiation schedule. Hence, a total dose of 42 Gy in 10 fractions over 5 days achieved a BED value at 68.5 Gy for acute effects as erythema (α/β ratio equal to 8). In the present study, the 42 Gy step conferred higher risk of acute skin toxicities with 64% of grade 1–2 erythema and 23% of grade 1–2 dry desquamation. Others non invasive APBI studies reported lower rate of acute radio-induced effects as the authors used irradiation scheme with lower BED. For instance, Livi and colleagues described 5% of grade 1 and 0.8% grade 2 acute skin toxicities after IMRT-APBI delivering a total dose of 30 Gy in 5 fractions over one week (BED value at 44.2 Gy for acute effects) [14]. Other study reported similar results to those form Livi and colleagues, with 6% of grade 2 acute skin toxicities occurred after a total dose of 38 Gy in 10 BID fraction over 5 days (BED value of 58.8 Gy) [15]. Similarly, Vicini and colleagues observed 43% of mild erythema and 12% of mild dry desquamation after 3D-conformal APBI (total dose of 38.5 Gy/ 10f/ 5d, i.e. BED value of 59.8 Gy).

When BED values were estimated for late toxicities, low α/β ratio is usually used (α/β ratio = 2). Even though a longer median FU of this study is warranted to accurately assess the role of 42 Gy in 10 fractions in the development of late toxicities, grade 1–2 breast pain was observed in 70% of patients and all grade 3 late toxicities in 11.8% of patients (fibrosis, telangiectasia, severe breast pain; n = 3) for an estimated BED value at 132 Gy. In recent report of Vicini and colleagues [16,4]; the authors described 30.8% of grade 1–2 breast pain and 4% of grade 3 late toxicities for a BED value estimated at 112.6 Gy.

Most of APBI clinical trials or prospective studies used an external beam irradiation (3D-conformal or intensity-modulated radiotherapy) with a total dose of 38.5 Gy and attempted to determine dosimetric factors predicting late toxicity occurrence. Despite treatment planning and delivery were conform to the National Surgical Adjuvant Breast and Bowel Project B-39/Radiation Therapy Oncology Group 0413 protocol (NSABP-B39/ RTOG 0413), none risk factor was retrieved. Hence Hepel and colleagues have initially described the maximum dose within the breast, PTV_EVAL and the breast volume exposure in proportion to the overall nontarget breast volume (V5–80) as risk factors of late toxicities occurrence (median follow-up of 15 months) [17]. At the opposite, recent initial results of RTOG 0319 did not get any risk factors of late toxicities after a longer median follow-up (36 months) [18]. Here, using a single institution experience and using the same 3D-conformal APBI technique, we showed through a prospective clinical trial that increasing total dose to a 42 Gy/ 10 fractions, BID could be a risk factor of early toxicities such as grade ≥2 dry and moist skin desquamation and of breast pain.

## Conclusions

Optimal total dose and fractionation are still debated even though most of APBI protocols use treatment planning and delivery in accordance to the NSABP-B39/ RTOG 0413 protocol. The total dose of 42 Gy in 10 fractions over 10 days is probably not optimal and can cause significant acute toxicity. Optimal total dose for efficacy and for tolerance would be determined through a larger cohort within a dose escalation phase II trial with mature data [19] and a longer follow-up is warranted to prospectively assess long-term toxicity.

## Competing interests

The authors declare that they have no competing interests.

## Authors’ contributions

CB was responsible of the phase II study and wrote the manuscript. All authors researched data for the article, provided substantial contributions to the discussion of content, and reviewed or edited the manuscript before submission. All authors read and approved the final manuscript.
